# Chromosome organization by fine-tuning an ATPase

**DOI:** 10.1101/gad.350627.123

**Published:** 2023-04-01

**Authors:** Lucia F. Massari, Adele L. Marston

**Affiliations:** The Wellcome Centre for Cell Biology, Institute of Cell Biology, School of Biological Sciences, University of Edinburgh, Edinburgh EH9 3BF, United Kingdom

**Keywords:** ATPase, acetylation, cohesin, cohesion, ECO1, ESCO1, NIPBL, SCC2, SMC

## Abstract

Cohesin is an ATPase that drives chromosome organization through the generation of intramolecular loops and sister chromatid cohesion. Cohesin's ATPase is stimulated by Scc2 binding but attenuated by acetylation of its Smc3 subunit. In this issue of *Genes & Development*, Boardman and colleagues (pp. 277–290) take a genetic approach to generate a mechanistic model for the opposing regulation of cohesin's ATPase by Scc2 and Smc3 acetylation. Their findings provide in vivo insight into how this important genome organizer functions in vivo.

## Abstract (precis)

In this Outlook, Massari and Marston discuss a study in this issue of *Genes & Development* from Boardman et al. that provides mechanistic insight into how cohesin's ATPase function is regulated by topological interactions between its components, Smc1 and Smc3, and auxiliary protein Scc2.

The four-subunit cohesin complex organizes chromosomes by generating intramolecular loops and sister chromatid cohesion. Two rod-shaped structural maintenance of chromosome (SMC) proteins, Smc1 and Smc3, bind each other at their extremities, forming a hinge at one end and an ATPase at the other through engagement of their head domains. The kleisin Scc1 connects the SMC heads, and Scc3 binds to Scc1. Both the association of cohesin with DNA and loop extrusion rely on cohesin undergoing cycles of ATP hydrolysis that drive the sequential association and release of DNA. Cohesin's ATPase was known to be positively regulated by the Scc2 protein and negatively regulated by the acetylation of Smc3, but how these opposing effects function in vivo was not well understood. Boardman et al. (2023) used budding yeast, combining suppressor screens, systematic analysis of point mutations, modeling using existing cohesin structures and functional assays in vivo to investigate the regulation of cohesin's ATPase activity. The findings lead to a molecular model that can explain how Scc2 and Smc3 acetylation influences cohesin's ATPase (Fig. 1).

**Figure 1.**
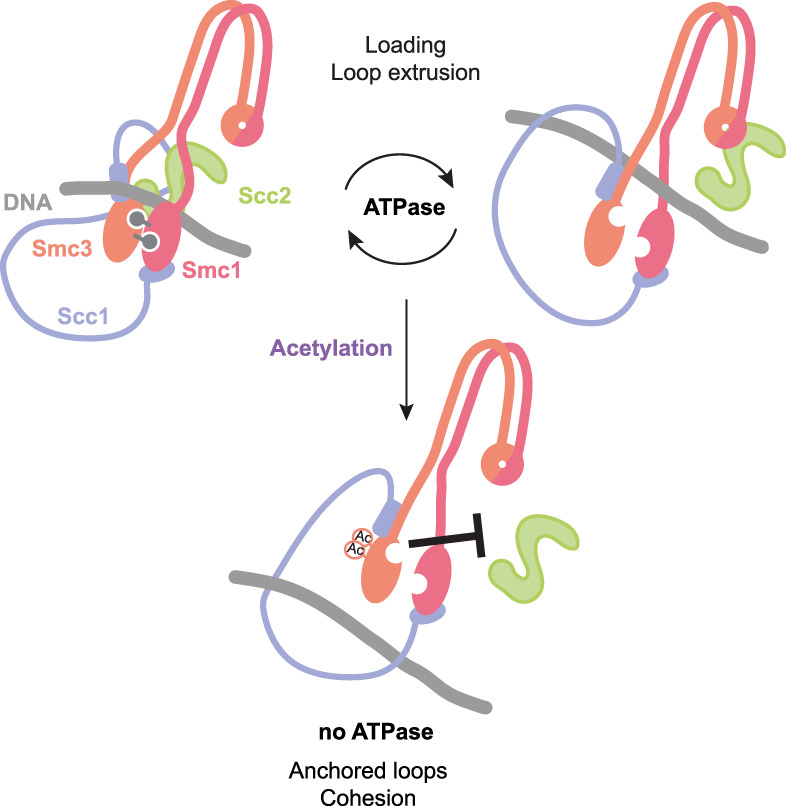
Smc3 acetylation opposes Scc2-dependent stimulation of cohesin's ATPase. Cycles of Scc2-stimulated ATP hydrolysis drive cohesin loading and loop extrusion. Smc3 acetylation blocks loading and loop extrusion, thereby anchoring loops and stabilizing cohesion. Boardman et al. (2023) provide genetic evidence that Scc2 modulates ATPase activity through interactions with Smc1 and that these interactions are blocked by Smc3 acetylation. Note that Scc3 is not shown for simplicity.

The Scc2 protein is essential both for cohesin loading onto DNA and loop extrusion (for review, see Davidson and Peters 2021). Scc2 stimulates cohesin's ATPase in vitro, and ATPase activity is required for both loading and loop extrusion (Davidson and Peters 2021). Structural studies showed that Scc2 contributes to formation of the clamp structure that blocks DNA on top of SMC heads (Collier et al. 2020).

Cohesin is also regulated by acetylation on its Smc3 subunit at residues K112 and K113 (in budding yeast) by the Eco1 acetyltransferase (Ben-Shahar et al. 2008; Ünal et al. 2008). Acetylation takes place once cohesin is loaded onto DNA and is necessary to establish cohesion because it counteracts cohesin release from DNA, mediated by Wpl1 (Ben-Shahar et al. 2008; Ünal et al. 2008). Recent studies have shown that Smc3 acetylation also restricts loop extrusion (Bastié et al. 2022) and is incompatible with cohesin loading. Substitution of acetyl-receptor lysines K112 and K113 on Smc3 with the acetyl-mimicking amino acid glutamine abolishes cohesin loading onto DNA (Hu et al. 2015) and reduces Scc2 stimulation of ATP hydrolysis in vitro (Murayama and Uhlmann 2015). Moreover, structural studies have shown that Scc2 binds to Smc3 on a surface containing K112 and K113 and that acidic residues on Scc2 form salt bridges with the positively charged K112 and K113 (Collier et al. 2020). Since acetylation neutralizes the positive charges of lysine residues, it is reasonable to think that it would weaken the interaction of Scc2 with cohesin.

Boardman et al. (2023) build from this observation to investigate how acetylation affects cohesin interaction with Scc2 in vivo and collect a series of findings all supporting the hypothesis that acetylation directly decreases Scc2 interaction with SMC heads. First, they characterized the acetyl-mimicking *smc3-K113Q* mutation: Consistent with previous work, they found that it is not viable. Inducible expression of Smc3-K113Q as sole source of Smc3 causes loss of cohesin binding to chromosomes and abolishes cohesion. Interestingly, all defects are at least in part rescued by Scc2 overexpression.

Next, screening for suppressor mutations of *smc3-K113Q*, they identified *smc1-T1117I*, which fully rescues *smc3-K113Q* viability and restores cohesion and cohesin loading on chromosomes. Smc1-T1117 sits between residues potentially involved in the Smc1/Scc2 interaction and a residue (K1121) important for ATP binding. Boardman et al. (2023) speculated that this position might be important for transmitting Scc2 binding to the ATPase site and promoting hydrolysis. If Scc2 binding is affected by acetylation or Smc3-K113Q, it might not be able to promote ATP hydrolysis. On the other hand, Smc1-T1117I substitution might on its own provide the structural shift that allows ATP hydrolysis in the absence of Scc2. To take this idea further, the investigators tested two predictions. First, only amino acid substitutions at Smc1-T1117 that cause a subtle change in the ATPase site to promote its activity would be viable and able to suppress Smc3-K113Q. Second, it should be possible to make other substitutions that decrease Scc2-mediated ATPase activity and thereby rescue the Smc3 nonacetylatable mutant *smc3-K113R*. Indeed, after testing all possible amino acid substitutions at this site, they only identified one other residue (valine, structurally similar to isoleucine) capable of rescuing *smc3-K113R*. Strikingly, they found that substitution to tryptophan (*smc1-T1117W*) instead rescues viability and cohesion of *smc3-K113R* cells.

Finally, Boardman et al. (2023) found further support for their model with the demonstration that the in vitro ATPase activity of Smc3-K113Q complexes cannot be stimulated by Scc2, but that Smc1-T1117I causes an increase in ATPase activity. In contrast, Smc1-T1117W displays lower activity. Overall, their findings suggest that acetylation blocks Scc2 interaction with cohesin head domains, preventing ATP hydrolysis and stopping the loop extrusion cycle. Indeed, recent work parallel to that of Boardman et al. (2023) uses an elegant in vivo cross-linking approach to identify interactions between Scc2 and SMC heads in different conformations, directly showing that Scc2 binding to Smc3 heads is blocked by acetylation (Kaushik et al. 2022).

These studies focus on the role of Smc3 acetylation in blocking access of Scc2; however, this is not the full story of how acetylation impacts cohesin function. Scc2 binding to cohesin is mutually exclusive with Pds5, and Pds5-bound cohesin does not have ATPase activity (Petela et al. 2018). Pds5 is essential for cohesion, negatively impacts loop extrusion (Bastié et al. 2022), and binds Smc3 on residues K112 and K113 (Petela et al. 2021). Pds5 may therefore specifically recognize cohesin acetylated on Smc3 to stabilize it on chromosomes. Understanding how the interchange between Scc2 and Pds5 regulates the molecular properties of cohesin to extrude loops and confer cohesion is an important question for the future.
